# Facility staffing associated with potentially avoidable hospitalizations in nursing home residents in Japan: a retrospective cohort study

**DOI:** 10.1186/s12877-023-04278-2

**Published:** 2023-09-15

**Authors:** Yoko Hamasaki, Nobuo Sakata, Xueying Jin, Takehiro Sugiyama, Kojiro Morita, Kazuaki Uda, Shinya Matsuda, Nanako Tamiya

**Affiliations:** 1 Graduate School of Comprehensive Human Sciences, University of Tsukuba, Tsukuba, Ibaraki Japan; 2 Health Services Research and Development Center, University of Tsukuba, Tsukuba, Ibaraki Japan; 3 Department of Health Services Research, Institute of Medicine, University of Tsukuba, 1-1-1 Tenno-Dai, Tsukuba, Ibaraki 305-8575 Japan; 4 Heisei Medical Welfare Research Institute, Shibuya-ku, Tokyo Japan; 5 Department of Social Science, Research Institute, National Center for Geriatrics and Gerontology, Obu, Aichi Japan; 6 Diabetes and Metabolism Information Center, Research Institute, National Center for Global Health and Medicine, Shinjuku-ku, Tokyo Japan; 7 Institute for Global Health Policy Research, Bureau of International Health Cooperation, National Center for Global Health and Medicine, Shinjuku-ku, Tokyo Japan; 8 Global Nursing Research Center, Graduate School of Medicine, University of Tokyo, Bunkyo-ku, Tokyo Japan; 9 Department of Preventive Medicine and Community Health, School of Medicine, University of Occupational and Environmental Health, Kitakyushu, Fukuoka Japan

**Keywords:** Potentially avoidable hospitalizations, Nursing homes, Japan, Long-term care

## Abstract

**Background:**

Wide variations in facility staffing may lead to differences in care, and consequently, adverse outcomes such as hospitalizations. However, few studies focused on types of occupations. Therefore, we aimed to examine the association between a wide variety of facility staffing and potentially avoidable hospitalizations of nursing home residents in Japan.

**Methods:**

In this retrospective cohort study using long-term care and medical insurance claims data in Ibaraki Prefecture from April 2018 to March 2019, we identified individuals aged 65 years and above who were newly admitted to nursing homes. In addition, facility characteristic data were obtained from the long-term care insurance service disclosure system. Subsequently, we conducted a multivariable Cox regression analysis and evaluated the association between facility staffing and potentially avoidable hospitalizations.

**Results:**

A total of 2909 residents from 235 nursing homes were included. The cumulative incidence of potentially avoidable hospitalizations at 180 days was 14.2% (95% confidence interval [CI] 12.7–15.8). Facilities with full-time physicians (adjusted hazard ratio [HR]: 0.59, 95% CI: 0.37–0.94) and a higher number of dietitians (HR: 0.72, 95% CI: 0.54–0.97) were significantly associated with a lower likelihood of potentially avoidable hospitalizations. In contrast, having nurses or trained caregivers during the night shift (HR: 1.72, 95% CI: 1.25–2.36) and a higher number of care managers (HR: 1.37, 95% CI: 1.03–1.83) were significantly associated with a high probability of potentially avoidable hospitalizations.

**Conclusions:**

We revealed that variations in facility staffing were associated with potentially avoidable hospitalizations. The results suggest that optimal allocation of human resources, such as dietitians and physicians, may be essential to reduce potentially avoidable hospitalizations. To provide appropriate care to nursing home residents, it is necessary to establish a system to effectively allocate limited resources. Further research is warranted on the causal relationship between staff allocation and unnecessary hospitalizations, considering the confounding factors.

**Supplementary Information:**

The online version contains supplementary material available at 10.1186/s12877-023-04278-2.

## Background

Japan introduced the long-term care (LTC) insurance system in 2000 to provide community- and facility-based care for responding to the needs of a super-aging society with the highest global percentage of people aged 65 and over [1]. Accordingly, the number of residents of LTC facilities for older adults in Japan is continuously increasing [2].

Compared to older adults living in their own homes, older adult residents of nursing homes experience more frequent hospitalizations [3], and 15.1–47.5% of them are hospitalized within one year after nursing home admission [3–7]. Hospitalizations of frail older adults are often related to negative events, such as loss of functional ability, delirium, hospital-acquired infections [8], and discontinuity of medications [9]. As hospitalizations may have more disadvantages than advantages for some facility residents, it is better to reduce their occurrence. In fact, some hospitalizations have been identified as potentially avoidable by providing appropriate preventive care or effective management [7]. Furthermore, medical costs for those aged 65 and over in Japan continue to increase, with more than half of these costs related to inpatient care [10]. A U.S. national study reported that 45% of hospitalizations of nursing home residents ($2.7 billion per year) were due to potentially avoidable hospitalizations (PAHs) [6], and this could be an important issue in Japan, as well.

Previous studies on nursing homes have reported that facility characteristics are associated with hospitalizations. Several studies in the U.S. have reported a lower likelihood of hospitalizations in nursing homes that have physician extenders (i.e., nurse practitioners or physician assistants) [5, 11], and special care units (e.g., those for dementia) [12]. However, most previous research is limited to residents with dementia [12] or outcome settings, such as hospitalization for infection [13] or hospitalization at the end of life [11, 12].

Nursing homes in Japan have been reported to possess a wide variety of facility characteristics [14]. While the Ministry of Health, Labour and Welfare (MHLW) has established several facility regulations, including minimum standards for facility staffing, several facilities are, in fact, additionally staffed [14, 15] with considerable variations in staffing. According to prior studies, these variations are considered to lead to differences in care and could also be related to the prognosis of residents, such as hospitalizations. Although few studies have focused on the types of occupations, such as physician and physician extenders, they did not consider other professionals, such as dietitians. Thus, in this study, we aimed to examine the association between a wide variety of facility staffing and PAHs of nursing home residents in Japan.

## Methods

### Setting

Three types of facility-based care services are provided under the Japanese LTC insurance system: nursing homes, geriatric intermediate care facilities, and medical LTC sanatoriums [1, 16]. Nursing homes are facilities for people whose medical conditions are stable but who need regular nursing care and who may reside permanently. In this study, we focused on nursing homes, which have the largest number of users (60.2% of the total residents) of the three types of facility-based care services in Japan [17].

Under the LTC insurance system regulations, each nursing home must be staffed with at least 3 nurses, 33 caregivers (including nurses), 1 dietitian, and 1 care manager (professionals responsible for planning and coordinating care or services for older people but not requiring a medical professional license) per 100 residents [18]. Physicians must be staffed in the required number and do not have to be full-time; part-time work is permitted.

### Study design and data source

This is a retrospective cohort study using medical insurance claims data, LTC insurance claims data, and LTC insurance service disclosure system data of Ibaraki Prefecture, Japan between April 2018 and March 2019. Data were collected by the local government and unique identifiers of the recipients and facilities were used to merge these databases. The population of Ibaraki Prefecture was 2,868,000 as of October 2018, of which 29.4% were aged 65 or older and 14.5% were aged 75 or older, a characteristic close to the national average (28.4% aged 65 or older and 14.7% aged 75 or older).

The medical insurance claims data include medical diagnosis and type of medical visit (i.e., hospitalization or outpatient care) [19]. The LTC insurance claims data include information regarding LTC residents, such as sex, age, care need level (CNL), location before facility admission, types of services provided, and dates of admission to or discharge from a nursing home [20]. If the residents die in the nursing home, their date of death is also recorded.

The LTC insurance service disclosure system has been used to identify facility-level characteristics. The system was established by the MHLW and administered by prefectural governments in accordance with the provisions of the LTC Insurance Law [21, 22]. The system includes information on staff members by occupation, such as physicians, nurses, dietitians, and other facility characteristics. We acquired data from the Healthcare Market Analysis Platform [23].

### Study population

Using resident-level LTC insurance data of Ibaraki Prefecture, we included individuals who were (1) aged 65 years and above and (2) newly admitted to nursing homes from July 2018 to March 2019. Individuals whose claims data were not linked to other data, those with missing data, and whose length of stay in the facility was less than three days were excluded. As data on newly opened facilities in the LTC insurance service disclosure system were unreliable, we also excluded the data of those admitted to facilities that had been in operation for less than one year.

### Outcome and follow-up

The outcome was 180-day PAHs. We followed the definition widely used for LTC residents, which was developed in a study funded by the Centers for Medicare and Medicaid Services, part of the U.S. Department of Health and Human Services [6, 24]. The study identified 16 condition groups, such as lower respiratory pneumonia or bronchitis, congestive heart failure, and urinary tract infection, which are considered to be potentially preventable or manageable in nursing homes. We re-categorized the conditions into 15 groups using the International Classification of Diseases, Tenth Revision (ICD-10) codes of Japan. When an individual had two or more diagnoses, we used only the primary diagnosis to identify the ICD-10 codes for categorization (see Additional Table 1 for a full list of conditions and ICD-10 codes).

Residents were followed from the date of their first nursing home admission between July 2018 and March 2019, referred to as the index date. For each outcome, residents were followed up with for 180 days until either the record of PAHs and hospitalizations other than PAHs, death, discharge from the nursing home after nursing home admission, or March 31, 2019 (end of the study period), whichever occurred first. Hospitalizations occurring within three days of discharge from a nursing home were included; this was considered to be related to nursing home stays [11, 25, 26], and residents who were hospitalized and subsequently died were treated as hospitalized.

### Independent variables of primary interest

The independent variable was facility characteristic pertaining to staffing. We obtained the full-time equivalent number of staff for physicians, nurses, caregivers, dietitians, care managers, and office workers. In Japanese nursing homes, the minimum number of staff required for each occupation is set by the MHLW according to the number of residents, and this is often described as the number of full-time equivalent staff per 100 beds in previous studies [4, 13, 15, 16]. Each staffing variable was categorized into three groups by tertiles (low, medium, and high) according to the distribution of staff numbers by facility level. The following categorical variables were also included: additional staffing of specific professionals (no additional staffing of specific professionals, presence of a full-time physician, and presence of nurses or trained caregivers during the night shift). This categorical variable was used because there were no facilities in which both types of professionals worked.

### Covariates

Based on previous studies, we considered the following individual and facility characteristics other than staffing as potential confounding factors: age [7, 27] (≤ 79 years, 80–89 years, and ≥ 90 years), sex [7], income level [5], location before facility admission [7], CNL [7], the Charlson Comorbidity Index (CCI) [6, 27], the number of beds in the facility [6, 27], and the facility’s number of years in business.

Income level, location before facility admission, and CNL were assigned using data recorded at the time point closest to the index date. Income level was identified from the record of copayment rate and categorized into two groups (i.e., ≤ 10% copayment for low to middle income, and 20% or 30% copayment for high income). The location before facility admission was classified as home, hospital, other LTC facilities, or others. CNL is a nationally standardized certification that incorporates an individual’s physical and mental status and ranges from 1 (least disabled) to 5 (most disabled) [28]. In general, people with CNL 1 have declining ability to engage in instrumental activities of daily living (IADLs). People with CNL 2 require partial assistance with activities of daily living (ADLs). People with CNL 3 have a significant decline in both IADLs and ADLs and require overall assistance. People with CNL 4 have a further decline in their functional ability and cognitive function, such as difficulty in standing or sitting, making it difficult for them to spend their daily lives without assistance. Finally, people with CNL 5 are mostly bedridden and have difficulty communicating and require overall assistance with IADLs and ADLs to perform their daily activities. The certificate of CNL is updated every 12 months in principle or whenever people experience functional changes. We re-categorized CNLs into three groups: lower-need level (CNLs 1–2), middle-need level (CNLs 3), and higher-need level (CNLs 4–5). The CCI is widely used to weigh the burden of comorbidities in administrative claims database research [29]. We calculated the CCI using an updated version of Quan’s protocol, which has been validated for use with Japanese administrative data [30], based on ICD-10 codes within three months before the index date. We categorized the CCI into four groups (0, 1–2, 3, and ≥ 4) [31].

### Statistical analysis

First, we described the baseline characteristics of the residents and facilities using median or percentages. Next, we estimated the cumulative incidence of PAHs and other events (occurrence of hospitalization other than PAH, death, or discharge to other places) using the cumulative incidence function [32]. We also described the patient’s diagnosis at the time of hospitalization. Then, we conducted a univariate analysis to identify the variables that are significantly associated with the outcome. Finally, we evaluated the associations between facility staffing and PAHs using a multivariate Cox regression model with adjustments for all the covariates mentioned earlier, representing resident and facility characteristics. These covariates were based on previous literature or the statistical significance of *p* < 0.10 in the univariate analysis. We used cluster robust standard errors to account for within-facility correlations and estimated adjusted hazard ratios for PAH in which the occurrence of other events (hospitalization other than PAH, death, or discharge to other places) were censored [33]. All data management and statistical analyses were conducted using Stata/MP version 15 (Stata Corp, College Station, TX, USA), with *p* < 0.05 (two-sided) denoting statistical significance.

As part of the post-hoc analysis, we explored whether the associations between facility staffing and PAHs were different by individual resident characteristics (CNL, CCI, and location before facility admission) using the interaction term.

## Results

### Study population and baseline characteristics of residents and facilities

Of the 3740 residents aged 65 and above admitted to nursing homes between July 1, 2018 and March 31, 2019, 2909 residents from 235 facilities were included in the analyses. We excluded 641 people whose claims data were not linked to other data, 70 people whose length of stay at the facility was three days or fewer, and 120 people admitted to facilities that had been in business for less than one year.

Tables 1 and 2 show the baseline characteristics of the residents and facilities, respectively. The median baseline age was 88 years (interquartile range [IQR] 84–92), 28.7% were male, and median follow-up period was 95 days (IQR 41–167). On admission to the facility, 42.4% of the residents were at CNL4. With respect to CCI, over 80% residents had a score of ≥ 1 (34.5% [*n* = 1003] with 1–2; 11.2% [*n* = 325] with 3; and 36.9% [*n* = 1073] with ≥ 4). For facility staffing that was not regulated by the law, only 1.3% and 5.5% of facilities had a full-time physician and nurses/trained caregivers during the night shift, respectively. The median number of residents per facility was 11 (IQR 8–16), with a minimum of 1 and a maximum of 33.

**Table 1:** Baseline characteristics of the residents in nursing homes (*N*=2909)

Characteristics	Median or n	IQR or %
Age (years)	88	(84–92)
Age group		
≤ 79	329	11.3
80–89	1359	46.7
≥ 90	1221	42.0
Sex (male)	836	28.7
Income level		
Low-Middle	2750	94.5
High	159	5.5
Care need level		
1	15	0.5
2	55	1.9
3	858	29.5
4	1234	42.4
5	747	25.7
Charlson comorbidity index		
0	508	17.5
1–2	1003	34.5
3	325	11.2
≥ 4	1073	36.9
Location before facility admission		
Home	1067	36.7
Hospital	737	25.3
Another long-term care facility	808	27.8
Other	297	10.2

Data are median (IQR) or n (%)

*IQR* Interquartile range

**Table 2:** Baseline characteristics of nursing homes (*N*=235)

Characteristics	Median or n	IQR or %
General characteristics		
Number of beds, Median (IQR)	50	50–70
Number of beds		
Low (< 51.0)	125	53.2
Medium (51.0–71.0)	52	22.1
High (> 71.0)	58	24.7
Years in business, Median (IQR)	15.7	10.7–24.3
Business years		
Short (< 13.0)	79	33.6
Medium (13.0–21.1)	78	33.2
Long (> 21.1)	78	33.2
Staffing characteristics		
Presence of a full-time physician	3	1.3
Presence of nurses or trained caregivers during night shift^a^	13	5.5
Number of total staff per 100 beds^b^		
Low(< 64.0)	71	30.2
Medium(64.0–76.6)	82	34.9
High(> 76.6)	82	34.9
Number of physicians per 100 beds^b^		
Low(< 0.19)	74	31.5
Medium(0.19–0.28)	81	34.5
High(> 0.28)	80	34.0
Number of nurses per 100 beds^b^		
Low(< 5.35)	53	22.6
Medium(5.35–6.6)	103	43.8
High(> 6.6)	79	33.6
Number of caregivers per 100 beds^b^		
Low(< 42.1)	77	32.8
Medium(42.1–51.7)	82	34.9
High(> 51.7)	76	32.3
Number of dietitians 100 beds^b^		
Low(< 1.6)	81	34.5
Medium(1.6–2.1)	91	38.7
High(> 2.1)	63	26.8
Number of care managers 100 beds^b^		
Low(< 1.5)	89	37.9
Medium(1.5–2.0)	95	40.4
High(> 2.0)	51	21.7
Number of office workers 100 beds^b^		
Low(< 3.1)	82	34.9
Medium(3.1–4.9)	78	33.2
High(> 4.9)	75	31.9

Data are median (IQR) or n (%)

*IQR* interquartile range

^a^Having night shift staff of nurses or caregivers who are capable of performing certain medical procedures

^b^Staffing variables were standardized to the total number of full-time equivalent staff per 100 beds and categorized into tertiles

### PAHs and other events

**Fig. 1:**
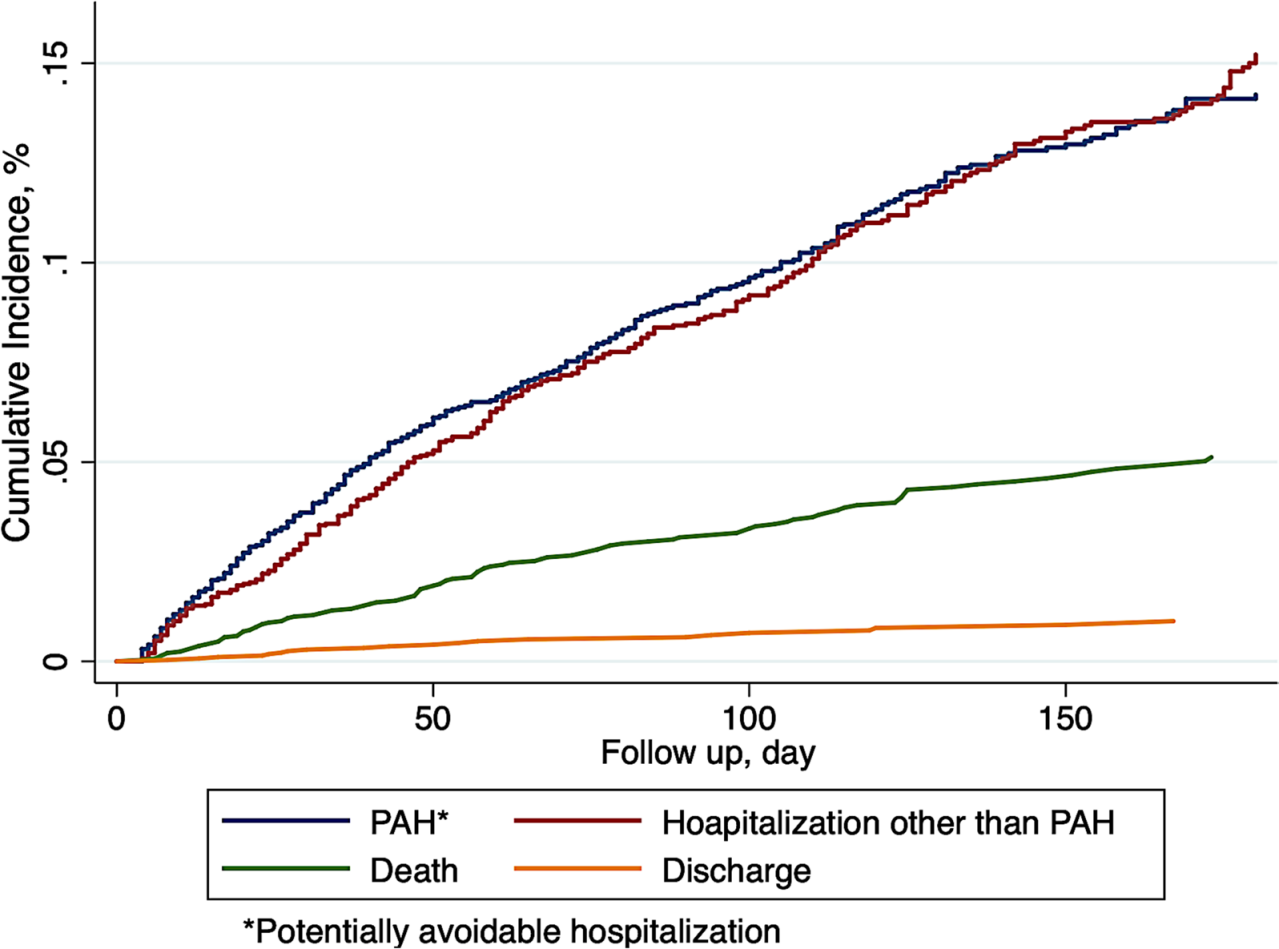
Cumulative incidences of potentially avoidable hospitalizations and other events (*N* = 2909)

A total of 302 (10.4%) residents experienced PAHs within the 180-day follow-up period. Figure 1 shows the cumulative incidence of PAHs and other events during the 180-day follow-up period. The cumulative incidence of PAHs at 180 days was 14.2% (95% CI 12.7–15.8). Regarding patients’ diagnosis at the time of hospitalization, five conditions (i.e., lower respiratory pneumonia or bronchitis, hypertension or hypotension, congestive heart failure, falls or trauma, and urinary tract infection) were responsible for 79.5% [240/302] of the total hospitalizations (an additional file shows this in more detail (see Additional Table 2)).

### Facility staffing associated with PAHs

Table 3 shows the unadjusted hazard ratios for PAHs. Table 4 shows the adjusted hazard ratios for PAHs. Multivariate Cox regression analysis revealed that facilities with a higher number of dietitians (compared with those with a lower number) and those with full-time physicians were significantly associated with a lower likelihood of PAHs, with adjusted hazard ratios of 0.72 (95% CI: 0.54–0.97) and 0.59 (95% CI: 0.37–0.94), respectively. In contrast, facilities with a higher number of care managers (compared with those with a lower number) and those with nurses or trained caregivers during the night shift were significantly associated with a higher probability of PAHs, with adjusted hazard ratios of 1.37 (95% CI: 1.03–1.83) and 1.72 (95% CI: 1.25–2.36), respectively. From the post-hoc analysis, the associations between facility staffing and PAHs were not different by individual resident characteristics (CNL, CCI, and location before facility admission) using the interaction term.

**Table 3:** Unadjusted hazard ratios for potentially avoidable hospitalization of resident and facility characteristics

Independent variables	Unadjusted hazard ratio (95% CI)		*p*-value
**Resident level**			
Age (Ref. ≤ 79)			
80–89	1.73	(1.09–2.77)	0.02
≥ 90	1.97	(1.26–3.10)	0.003
Sex			
Female (Ref. male)	0.75	(0.59–0.95)	0.02
Income level (Ref. Low–Middle)			
High	0.47	(0.23–0.95)	0.04
Care need level (Ref. 1–3)			
4	1.17	(0.89–1.53)	0.27
5	1.34	(0.97–1.84)	0.08
Charlson comorbidity index (Ref. 0)			
1–2	1.16	(0.78–1.72)	0.45
3	1.57	(0.99–2.48)	0.05
≥ 4	1.85	(1.28–2.68)	0.001
Location before facility admission (Ref. home)			
Hospital	1.61	(1.24–2.11)	< 0.001
Another long-term care facility	0.87	(0.65–1.17)	0.35
Other	0.80	(0.55–1.16)	0.24
**Facility Level**			
Business years (Ref. short (< 13.0))			
Medium (13.0–21.1)	1.28	(1.02–1.61)	0.03
Long (> 21.1)	0.99	(0.74–1.35)	0.996
Number of beds (Ref. low (< 51.0))			
Medium (51.0–71.0)	1.18	(0.90–1.54)	0.24
High (> 71.0)	1.14	(0.87–1.48)	0.34
Additional staffing of specific professionals (Ref. no additional staffing)			
Presence of a full-time physician	0.66	(0.46–0.97)	0.03
Presence of nurses or trained caregivers during night shift^a^	1.65	(1.23–2.21)	0.001
Number of physicians per 100 beds^b^ (Ref. low (< 0.19))			
Medium (0.19–0.28)	0.90	(0.67–1.21)	0.48
High (> 0.28)	0.97	(0.76–1.25)	0.82
Number of nurses per 100 beds^b^ (Ref. low (< 4.9))			
Medium (4.9–6.6)	0.99	(0.76–1.30)	0.97
High (> 6.6)	1.03	(0.77–1.38)	0.82
Number of caregivers per 100 beds^b^ (Ref. low (< 42.1))			
Medium (42.1–51.7)	0.88	(0.66–1.18)	0.39
High (> 51.7)	1.05	(0.81–1.37)	0.72
Number of dietitians per 100 beds^b^ (Ref. low (< 1.6))			
Medium (1.6–2.1)	0.86	(0.67–1.10)	0.22
High (> 2.1)	0.75	(0.55–1.01)	0.06
Number of care managers per 100 beds^b^ (Ref. low (< 1.5))			
Medium (1.5–2.0)	0.99	(0.77–1.27)	0.93
High (> 2.0)	1.23	(0.93–1.64)	0.15
Number of office workers per 100 beds^b^ (Ref. low (< 3.1))			
Medium (3.1–4.9)	1.17	(0.89–1.54)	0.26
High (> 4.9)	1.03	(0.79–1.34)	0.84

*CI* Confidence interval

^a^Having night shift staffing of nurses or caregivers who are capable of performing certain medical procedures

^b^Staffing variables were standardized to the total number of full-time equivalent staff per 100 beds and categorized into tertiles

**Table 4:** Adjusted hazard ratios for potentially avoidable hospitalization of staffing in nursing homes

Staffing characteristics	Adjusted hazard ratio (95% CI) ^a^		*p-*value
Additional staffing of specific professionals (Ref. no additional staffing)			
Presence of a full-time physician	0.59	(0.37–0.94)	0.03
Presence of nurses or trained caregivers during night shift^b^	1.72	(1.25–2.36)	0.001
Number of physicians per 100 beds^c^ (Ref. low (< 0.19)			
Medium (0.19–0.28)	1.08	(0.75–1.57)	0.67
High (> 0.28)	1.10	(0.84–1.45)	0.48
Number of nurses per 100 beds^b^ (Ref. low (< 4.9)			
Medium (4.9–6.6)	0.93	(0.69–1.23)	0.59
High (> 6.6)	1.07	(0.78–1.48)	0.68
Number of caregivers per 100 beds^b^ (Ref. low (< 42.1)			
Medium (42.1–51.7)	0.77	(0.58–1.01)	0.06
High (> 51.7)	1.00	(0.75–1.35)	0.98
Number of dietitians per 100 beds^b^ (Ref. low (< 1.6)			
Medium (1.6–2.1)	0.83	(0.62–1.09)	0.18
High (> 2.1)	0.72	(0.54–0.97)	0.03
Number of care managers per 100 beds^b^ (Ref. low (< 1.5)			
Medium (1.5–2.0)	1.05	(0.76–1.44)	0.79
High (> 2.0)	1.37	(1.03–1.83)	0.03
Number of office workers per 100 beds^b^ (Ref. low (< 3.1)			
Medium (3.1–4.9)	1.19	(0.91–1.55)	0.20
High (> 4.9)	1.20	(0.89–1.62)	0.23

*CI* Confidence interval

^a^Adjusted for age, sex, income level, location before facility admission, care need level, the Charlson Comorbidity Index, the number of beds in the facility, and the facility’s number of years in business

^b^Having night shift staffing of nurses or caregivers who are capable of performing certain medical procedures

^c^Staffing variables were standardized to the total number of full-time equivalent staff per 100 beds and categorized into tertiles

## Discussion

To the best of our knowledge, this is the first longitudinal study to identify the association between wide variations of facility staffing and PAHs of nursing home residents. We found that staffing of dietitians, care managers, full-time physicians, and a night staff of nurses or trained caregivers were associated with PAHs. Although some of the findings require caution in interpreting the results due to the influence of the distribution of staffing in the facilities, the association between dietitians and lower risk of PAH was particularly noteworthy.

Regarding the association between staffing of dietitians and outcomes for facility residents, a previous nationwide study of nursing homes in Japan suggested that registered dietitians play an important role in preventing CNL deterioration [15]. The current study found that the relative risk of PAHs was lower for residents of nursing homes with a higher number of dietitians than for those with a lower number of dietitians. Taken together, these results suggest that dietitians may contribute not only to maintaining physical performance but also to lowering the risk of PAHs.

With regard to care managers, the relative risk of PAHs was found to be higher in residents of nursing homes with a higher number of care managers compared to nursing homes with a lower number. To better interpret the results, we examined the interaction between care manager and resident characteristics; however, there was no statistically significant difference. Therefore, regardless of individual characteristics, facility characteristics were considered to be associated with PAH. Although no previous study is related to our findings, a possible reason for the results was the smooth collaboration between medical institutions. As care managers intervene in the process of selecting care services in the nursing home and coordinate services outside for the resident, they have a network of medical facilities. Therefore, a higher number of care managers in the nursing home might expand the choice of available medical facilities and facilitate the coordination of hospitalization. At the same time, nursing homes with residents with a high probability of hospitalization may have more care managers. Future studies are needed to clarify the role of care managers in nursing homes and should focus on care managers, adjusting for confounding factors.

With regard to the allocation of full-time physicians, the results of the multivariable analysis indicated a lower risk of PAHs. This result was similar to a previous study indicating that hospital-based facilities had a lower risk of PAHs; possibly due to better access to physicians [11]. One study in Japan found that the allocation of a full-time physician was associated with dying in a nursing home [34]. From our findings, obtaining routine physician care may help prevent unnecessary hospitalizations. However, in this study, only three facilities had full-time physicians, and our results may reflect the characteristics of each facility rather than the presence of full-time physicians. Therefore, the association from the present findings should be discussed with caution. Further research on the role played by full-time physicians in nursing homes with a larger sample size data set may be worth considering.

In contrast to full-time physicians, night staff of nurses or trained caregivers was associated with more PAHs. Nurses and trained caregivers working night shifts are expected to address medical needs, and facilities with night staff are likely to have more residents requiring medical procedures. For example, a previous study in Japanese nursing homes showed that the use of artificial nutrition is significantly associated with a higher risk of PAH and other hospitalizations [35]. Thus, our findings may reflect that confounding factors such as medical procedures were not fully adjusted for. Moreover, only 13 facilities in this study had night staff, and the present results may reflect the characteristics of each facility. Therefore, as with the results for full-time physicians, careful interpretation of the present findings is required. Further research controlling for confounding factors may reveal details of the relationship between night staff and hospitalization.

This study has several limitations. First, there may be residual confounders such as disease severity. Although we used the CCI, we could not acquire information on the use of medical procedures (e.g., oxygen therapy) or medications (e.g., a high number of medications). Previous studies have identified that these factors may influence the likelihood of hospitalization [7]. Second, the findings should be interpreted with caution because whether all PAHs can truly be avoided is likely to vary on a case-by-case basis. Decisions regarding hospitalization are complex, and the development of indicators that accurately distinguish between preventable and necessary hospitalizations is not a straightforward process [36]. To overcome these barriers, we used the definition of PAH developed by the Centers for Medicare and Medicaid Services, which has been widely used for residents of nursing homes [6, 11, 24]. Third, since this study was conducted in a single prefecture, regional differences in human resources or availability of home care could affect the generalizability of our results. However, the numbers of physicians, nurses, dietitians, caregivers, and care managers per LTC residents in Ibaraki were largely similar to those in Japan.

## Conclusions

This study revealed that variations in facility staffing were associated with PAHs among nursing home residents. The results suggest that optimal allocation of human resources, such as dietitians and physicians, may be essential for reducing PAHs. To provide appropriate care to nursing home residents, it is necessary to establish a system to effectively allocate limited resources. Further research is warranted on the causal relationship between staff allocation and unnecessary hospitalizations, considering the confounding factors.

### Supplementary Information

**Additional file 1: Additional Table 1.** International Classification of Diseases 10th Revision codes to assess ambulatory care sensitive conditions. **Additional Table 2.** Reasons for potentially avoidable hospitalization.

## Data Availability

The datasets for individual information generated and/or analyzed during the current study, which includes LTC insurance claims data and medical insurance claims data are not publicly available because the local government of Ibaraki Prefecture owns the original data and only approved the secondary use of the data for the current study. The data of each LTC facility is published on the website “Care Information Disclosure System”, (http://www.kaigokensaku.mhlw.go.jp). The aggregated data are available from the corresponding author on reasonable request with the permission of the local government of Ibaraki Prefecture.

## Abbreviations

CCI: Charlson Comorbidity Index
CI: Confidence interval
CNL: Care need level
HR: Adjusted hazard ratio
ICD-10: International Classification of Diseases, Tenth Revision
IQR: Interquartile range
LTC: Long-term care
PAH: Potentially avoidable hospitalization
