# Supplementary material for: Facility staffing associated with potentially avoidable hospitalizations in nursing home residents in Japan: a retrospective cohort study
Source: BMC Geriatr. 2023 Sep 15;23:566. doi: 10.1186/s12877-023-04278-2 (PMC10504825; doi:10.1186/s12877-023-04278-2)
Supplement: Supplementary file 1 — Additional file 1: Additional Table 1. International Classification of Diseases 10th Revision codes to assess ambulatory care sensitive conditions. Additional Table 2. Reasons for potentially avoidable hospitalization. [file 12877_2023_4278_MOESM1_ESM.docx]

Additional Table 1. International Classification of Diseases 10th Revision codes to assess ambulatory care sensitive conditions

| **Condition** | **Walsh et al. (2010) ^1^**  **ICD-9** | **Current paper**  **ICD-10** |
| --- | --- | --- |
| Anemia | 280.0, 280.1, 280.8, 280.9, 281.0-281.4, 281.8, 281.9, 285.21, 285.22, 285.29, 285.9 | D500, D501, D508, D509, D510, D511, D513, D518, D520, D521, D528, D529, D530-D532, D538, D539, D630, D638, D649 |
| Congestive heart failure | 398.91, 402.11, 402.91, 404.11, 404.13, 404.91, 404.93, 428.0, 428.1, 428.20-428.23, 428.30-428.33, 428.40-428.43, 428.9, 518.4 | I098, I110, I130, I132, I500, I501, I509, J81 |
| Hypertension and hypotension | 401.9, 402.10, 402.90, 403.10, 403.90, 404.10, 404.90 | Hypertension: I10, I119, I129, I131 |
|  | 458.0, 458.1, 458.21, 458.29, 458.8, 458.9 | Hypotension: I951, I952, I958, I959 |
| Poor glycemic control | 250.02, 250.03, 250.10-250.13, 250.20-250.23, 250.30-250.33, 251.0, 251.2, 790.29 | E10, E11, E131, E15, E162, R730 |
| Dehydration, volume depletion, Acute renal failure, Hyponatremia | Dehydration, volume depletion:276.5, 276.8 | Dehydration, volume depletion: E86, E876 |
|  | Acute renal failure: 584.5-584.9, 588.81, 588.89, 588.9 | Acute renal failure: N17, N170-N172, N178, N179, N258, N259 |
|  | Hyponatremia: 276.1 | Hyponatremia: E871 |
| Constipation, fecal impaction, obstipation | 560.39, 564.00, 564.01, 564.09 | K564, K590 |
| Diarrhea, gastroenteritis, *Clostridium Difficile* | 003.0, 004.0-004.3, 004.8, 004.9, 005.0-005.4, 005.81, 005.89, 005.9, 006.0, 007.0-007.5, 007.8, 007.9, 008.00-008.04, 008.09, 008.1-008.3, 008.41-008.44, 008.46, 008.47, 008.49, 008.5, 008.61-008.67, 008.69, 008.8, 009.0-009.3, 558.9, 787.91 | Diarrhea and gastroenteritis: A020, A030-A033, A038, A039, A040-A046, A048, A049, A050-A054, A058, A059, A060, A070-A073, A078, A079, A080-A083, A090, A099, K522, K523, K528, K529 |
|  | 008.45 | *Clostridium Difficile* : A047 |

Additional Table 1. (Continued)

| **Condition** | **Walsh et al. (2010) ^1^**  **ICD-9** | **Current paper**  **ICD-10** |
| --- | --- | --- |
| Skin ulcers, cellulitis | Skin ulcers: 707.00-707.07, 707.09, 707.10-707.15, 707.19, 707.8, 707.9 | Skin ulcers: L890-L893, L899, L97, L984 |
|  | Cellulitis: 681.00-681.02, 681.10, 681.11, 681.9, 682.0- 682.9, 683, 686.00, 686.01, 686.09, 686.1, 686.8, 686.9 | Cellulitis: L030-L033, L038, L039, L049, L080, L088, L089, L88, L980, E832 |
| Lower respiratory pneumonia and bronchitis | 480.0-480.3, 480.8, 480.9, 481, 482.0-482.2, 482.30-482.32, 482.39-482.41, 482.49, 482.81-482.84, 482.89, 482.9, 483.0, 483.1, 483.8, 485, 486,507.0 | J120-J122, J128, J129, J13, J14, J150-J159, J160, J168, J180, J189, J690, A481 |
| Urinary tract infection | 590.10, 590.11, 590.80, 590.81, 590.9, 595.0-595.2, 595.4, 595.89, 595.9, 597.0, 598.00, 598.01, 599.0, 601.0-601.4, 601.8, 601.9 | N10, N12, N159, N160-N165, N168, N300-N302, N308, N309, N340, N351, N37, N390, N410-N413, N418, N419, N51 |
| Falls and trauma | 800.00-800.06, 800.09-800.16, 800.19-800.26, 800.29-800.36, 800.39-800.46, 800.49-800.56, 800.59-800.66, 800.69-800.76, 800.79-800.86, 800.89-800.96, 800.99-801.06, 801.09-801.16, 801.19-801.26, 801.29-801.36, 801.39-801.46, 801.49-801.56, 801.59-801.66, 801.69-801.76, 801.79-801.86, 801.89-801.96, 801.99, 802.0, 802.1, 802.20-802.39, 802.4-802.9, 803.00-803.06, 803.09-803.16, 803.19-803.26, 803.29-803.36, 803.39-803.46, 803.49-803.56, 803.59-803.66, 803.69-803.76, 803.79-803.86, 803.89-803.96, 803.99-804.06, 804.09-804.16, 804.19-804.26, 804.29-804.36, 804.39-804.46, 804.49-804.56, 804.59-804.66, 804.69-804.76, 804.79-804.86, 804.89-804.96, 804.99 | Skull and face fractures: S020, S021, S022, S023, S024, S026, S029 |

Additional Table 1. (Continued)

| **Condition** | **Walsh et al. (2010) ^1^**  **ICD-9** | **Current paper**  **ICD-10** |
| --- | --- | --- |
| Falls and trauma (Continued) | 805.00-805.08, 805.10-805.18, 805.3, 805.5-805.7, 805.9, 806.00-806.39, 806.4, 806.5, 806.60-806.62, 806.69-806.72, 806.79, 806.8, 806.9, 807.00-807.19, 807.2-807.6, 808.0-808.3, 808.41-808.43, 808.49, 808.51-808.53, 808.59, 808.8,808.9, 809.0, 809.1, 810.00-810.03, 810.10-810.13, 811.00-811.03, 811.09-811.13, 811.19 | Other fractures: S120, S121, S122, 3S124, S125, S126, S128, S129, S220, S222, S223, S224, S225, S229, S320, S321, S322, S323, S324, S325, S326, S328, S329, S420, S421, S429 |
|  | 812.00-812.03, 812.09-812.13, 812.19-812.21, 812.30, 812.31, 812.40-812.44, 812.49-812.54, 812.59, 813.00-813.08, 813.10-813.18, 813.20-813.23, 813.30-813.33, 813.40-813.45, 813.50-813.54, 813.80-813.83, 813.90-813.93, 814.00-814.19, 815.00-815.04, 815.09-815.14, 815.19, 816.00-816.03, 816.10-816.13, 817.0, 817.1, 818.0, 818.1, 819.0, 819.1 | Fracture of upper limb: S422, S423, S424, S520, S521, S523, S524, S525, S536, S527, S529, S620, S621, S623, S625, S626, S629 |
|  | 822.0, 822.1, 823.00-823.02, 823.10-823.12, 823.20-823.22, 823.30-823.32, 823.40-823.42, 823.80-823.82, 823.90-823.92, 824.0-825.1, 825.20-825.25, 825.29-825.35, 825.39, 826.0, 826.1, 827.0, 827.1, 828.0, 828.1, 829.0, 829.1 | Fracture of lower limb: S723, S724, S729, S820, S821, S822, S823, S824, S825, S829, S920, S921, S922, S923, S924, S925, S928, S929 |
|  | 820.00-820.03, 820.09-820.13, 820.19-820.22, 820.30-820.32, 820.8, 820.9, 821.00, 821.01, 821.10, 821.11, 821.20-821.23, 821.29-821.33, 821.39 | Fracture of neck of femur (hip): S720, S721, S722 |

Additional Table 1. (Continued)

| **Condition** | **Walsh et al. (2010) ^1^**  **ICD-9** | **Current paper**  **ICD-10** |
| --- | --- | --- |
| Falls and trauma (Continued) | 830.0, 830.1, 831.00-831.04, 831.09-831.14, 831.19, 832.00-832.04, 832.09-832.14, 832.19, 833.00-833.05, 833.09-833.15, 833.19, 834.00-834.02, 834.10-834.12, 835.00-835.03, 835.10-835.13, 836.0-836.4, 836.50-836.54, 836.59-836.64, 836.69,  837.0, 837.1, 838.00-838.06, 838.09- 838.16, 838.19, 839.00-839.08, 839.10-839.18, 839.20, 839.21, 839.30, 839.31, 839.40-839.42, 839.49-839.52, 839.59, 839.61, 839.69, 839.71, 839.79, 839.8, 839.9 | Dislocation: S030, S131, S331, S332, S333, S430, S431, S432, S530, S531, S630, S631, S730, S830, S831, S832, S930, S931, S933 |
|  | 850.0, 850.11, 850.12, 850.2-850.5, 850.9, 851.00-851.06, 851.09-851.16, 851.19-851.26, 851.29, 851.31-851.36, 851.39-851.46, 851.49-851.56, 851.59-851.66, 851.69-851.76, 851.79-851.86, 851.89-851.96, 851.99, 852.00, 852.03, 852.04, 852.10, 852.12-852.16, 852.19-852.26, 852.29, 852.31-852.36, 852.39-852.46, 852.49-852.56, 852.59, 853.00, 853.03, 853.04, 853.09-853.16, 853.19, 854.00-854.06, 854.09-854.16, 854.19, 905.0-905.9, 907.0-907.5, 907.9, 908.0-908.6, 908.9, 909.0-909.5, 909.9 | Intracranial injury: S060, S061, S063, S064, S065, S066, S069 |
|  | 925.1, 925.2, 926.0, 926.11, 926.12, 926.19, 926.8, 926.9, 927.00-927.03, 927.09-927.11, 927.20, 927.21, 927.3, 927.8, 927.9, 928.00, 928.01, 928.10, 928.11, 928.20, 928.21, 928.3, 928.8, 928.9, 929.0, 929.9, | Crushing injury or internal injury: S049, S070, S079, S090, S098, S099, S119, S141, S142, S149, S179, S259, S269, S280, S298, S369, S379, S380, S381, S398, S449, S479, S489, S498, S570, S578, S598, S670, S672, S673, S698, S749, S77, S770, S771, S772, S798, S87, S870, S878, S898, S970, S971, S978 |

Additional Table 1. (Continued)

| **Condition** | **Walsh et al. (2010) ^1^**  **ICD-9** | **Current paper**  **ICD-10** |
| --- | --- | --- |
| Falls and trauma (Continued) | 851.30, 852.10, 852.30 | Open wounds of head, neck, and trunk: S014, S019, S211, S212, S310, S410, S411, S510, S710 |
|  |  | Open wounds of extremities: S614, S615, S910, S911, S913 |
|  | 906.0-906.9 | Superficial injury, contusion: S009 |
|  | 940.0-940.5, 940.9, 941.00-941.59, 942.00-942.05, 942.09-942.15, 942.19-942.25, 942.29-942.35, 942.39-942.45, 942.49-942.55, 942.59, 943.00-943.06, 943.09-943.16, 943.19-943.26, 943.29-943.36, 943.39-943.46, 943.49-943.56, 943.59-944.08, 944.10-944.18, 944.20-944.28, 944.30-944.38, 944.40-944.48, 944.50-944.58, 945.00-945.06, 945.09-945.16, 945.19-945.26, 945.29-945.36, 945.39-945.46, 945.49-945.56, 945.59, 946.0-946.5, 947.0-947.4, 947.8, 947.9, 948.00, 948.10, 948.11, 948.20-948.22, 948.30-948.33, 948.40, 948.44, 948.50-948.55, 948.60-948.66, 948.70-948.77, 948.80-948.88, 948.90-948.99, 949.0-949.5 | Burns T200, T201, T202, T203, T210, T211, T212, T213, T220, T221, T222, T223, T230, T231, T232, T233, T240, T241, T242, T243, T250, T251, T252, T253, T260, T261, T262, T264, T265, T266, T271, T280, T281, T282, T283, T284, T300, T310, T311, T312, T313, T314, T315, T316, T317, T318, T319 |
|  | 959.01, 959.09, 959.11-959.14, 959.19, 959.2-959.9, 991.6, 991.8, 991.9, 992.0-992.9, 994.1-994.8 | Other injuries & conditions due to external causes T07, T149, T159, T509, T659, T670, T671, T672, T673, T674, T675, T676, T677, T678, T679, T68, T698, T699, T711, T730, T731, T751, T754, T758, T788, T799, T889 |

Additional Table 1. (Continued)

| **Condition** | **Walsh et al. (2010) ^1^**  **ICD-9** | **Current paper**  **ICD-10** |
| --- | --- | --- |
| Altered mental status, acute confusion, delirium Psychosis, severe agitation, organic brain syndrome | Altered mental status, acute confusion, delirium: 290.3, 290.41, 292.81, 293.0, 293.1 | F010-F013 F018 F019 F03 F050 D051 F058 F059 F199 |
|  | Psychosis, severe agitation, organic brain syndrome: 290.42, 290.43, 290.8, 290.9, 293.81-293.84, 293.89, 293.9, 297.0-297.3, 297.8, 297.9, 298.0-298.4, 298.8, 298.9 | F01, F03, F060-F064, F068 F220, F228, F229 F230-F233, F238, F239, F24, F28, F29, F323, F333, F448, F530, F531, F538, F539 |
| Chronic-obstructive pulmonary disease, Asthma | Chronic-obstructive pulmonary disease: 466.0, 466.11, 466.19, 490, 491.0, 491.1, 491.20, 491.21, 491.8, 491.9, 492.0, 492.8, 494.0, 494.1, 496 | J200, J209, J218, J40, J410, J411, J418, J42, J439, J441, J449, J47 |
|  | Asthma: 493.00, 493.01, 493.02, 493.10, 493.11, 493.12, 493.20, 493.21, 493.22, 493.81, 493.82, 493.90, 493.91, 493.92, | Asthma: J440, J441, J449, J459 |
| Weight loss, nutritional deficiencies, seizures | Weight loss: 783.21, 783.22, 783.3, 783.7 | Weight loss: R633, R634, R636 |
|  | Nutritional deficiencies: 260, 261, 262, 263.0, 263.1, 263.2, 263.8, 263.9, 268.0, 268.1, | Nutritional deficiencies: E40, E41, E43, E440, E441, E45, E46, E550, E643 |

ICD-9=International Classification of Diseases, Ninth Revision (ICD-9) codes, ICD-10= International Classification of Diseases, Tenth Revision (ICD-10) codes

^1^ Walsh EG, Freiman M, Haber S, Bragg A, Ouslander J, JM W. Cost drivers for dually eligible beneficiaries: Potentially avoidable hospitalizations from nursing facility, skilled nursing facility and home and community-based services waivers programs. Final task 2 report. RTI International, Waltham, MA. 2010.

Additional Table 2. Reasons for potentially avoidable hospitalization

| **Rank** | **Condition^a^** | **n** | **%** |
| --- | --- | --- | --- |
| 1 | Lower respiratory pneumonia and bronchitis | 91 | 30.1 |
| 2 | Hypertension/Hypotension | 54 | 17.9 |
| 3 | Congestive heart failure | 42 | 13.9 |
| 4 | Falls and trauma | 31 | 10.3 |
| 5 | Urinary tract infection | 22 | 7.3 |
| 6 | Poor glycemic control | 9 | 3.0 |
| 7 | Dehydration, Acute renal failure, Hyponatremia | 9 | 3.0 |
| 8 | Constipation, Fecal impaction, Obstipation | 8 | 2.6 |
| 9 | Seizures | 8 | 2.6 |
| 10 | Chronic obstructive pulmonary disease, Chronic bronchitis, Asthma | 7 | 2.3 |
| 11 | Diarrhea, gastroenteritis, Clostridium difficile | 6 | 2.0 |
| 12 | Skin ulcers, Cellulitis | 5 | 1.7 |
| 13 | Altered mental status, Acute confusion, Delirium/Psychosis, Severe agitation, Organic brain syndrome | 5 | 1.7 |
| 14 | Weight loss, Nutritional deficiencies | 3 | 1.0 |
| 15 | Anemia | 2 | 0.7 |
|  | All | 302 | 100 |
| ^a^ We used primary diagnosis to identify the codes of the International Classification of Diseases, Tenth Revision (ICD-10 code) for categorization | | | |
